# Epithelioid Haemangioma of Bone: A Case Series and Comprehensive Literature Review Reappraising the Diagnostic Classification of All Epithelioid Vascular Neoplasms of Bone

**DOI:** 10.7759/cureus.15371

**Published:** 2021-06-01

**Authors:** Subramaniam Ramkumar

**Affiliations:** 1 Pathology, Woodland Hospital, Shillong, IND

**Keywords:** bone neoplasms, epithelioid, pseudomyogenic haemangioendothelioma, vascular neoplasms, bone, epithelioid haemangioma, epithelioid haemangioendothelioma, epithelioid angiosarcoma, fos gene, wwtr1–camta1

## Abstract

Epithelioid vascular neoplasms of the bone are classified by the World Health Organization (WHO) into only two tiers: low-grade epithelioid hemangioma (EH) and a more malignant category including both epithelioid hemangioendothelioma and epithelioid angiosarcoma. The World Health Organization defines bone EH as a locally aggressive neoplasm with no connotation of benign or intermediate malignancy. We reviewed three cases of EH in our lab archives with the perspective of appraising their histomorphological approach toward diagnosis. Patients were in the age range of 15-25 years. The site of the neoplasms ranged from the carpal bones to the metatarsal bones. Histomorphological examination of the lesions showed a nodular growth pattern of a vascular neoplasm without demonstrable vessel origin. The vasoformative area increased from the center to the periphery, with prominent epithelioid morphology of the endothelial cells at the periphery and an associated inflammatory infiltrate comprising eosinophils, lymphocytes, and plasma cells. The growth pattern was diffuse, with extension into the deeper dermis of overlying skin.

## Introduction

Vascular tumors encompass a wide histologic spectrum, including hemangioma, hemangioendothelioma, angiosarcoma, and respective epithelioid variants. The World Health Organization (WHO) classified soft-tissue epithelioid vascular tumors into three distinct entities based on malignancy level: benign epithelioid hemangioma (EH) [1], intermediate-grade epithelioid hemangioendothelioma (EHE) [1], and malignant epithelioid angiosarcoma (EAS). However, the same edition of WHO classifies bone epithelioid vascular tumors into only two levels: EH and a more malignant category including both EHE and EAS [1]. This revised classification of bone epithelioid vascular lesions is likely owing to reported EH cases with recurrence and lymph node involvement. The intermediate category of classifying soft-tissue epithelioid vascular neoplasms is defined by an infiltrative and locally destructive growth pattern, often recurring and occasionally (<2%) metastasizing [1]. If these criteria are applied to EH of bone, recurring in 11% and metastasizing in 2.7%, this entity fits best within this intermediate category, in between benign and angiosarcoma (malignant) of bone [2]. However, WHO defines bone EH as a locally aggressive neoplasm with no connotation of benign or intermediate malignancy. Therefore, EH continues to be confused with and erroneously misclassified as EHE or some other vascular sarcoma type. This terminology and classification have proven particularly controversial for intraosseous epithelioid vascular tumors at the low end of the malignancy spectrum [3-4]. Here, we describe the clinical and histomorphological features of three cases with osteolytic lesions in the limb extremities and re-evaluate the criteria for distinguishing EH from similar reactive and neoplastic epithelioid vascular lesions based on previous studies.

## Case presentation

Bone curettage specimens from three patients with bone EH were retrospectively analyzed.

EH diagnosis was established based on hematoxylin and eosin (H&E) and immunohistochemical (IHC) staining of paraffin-embedded tissue sections. In each biopsy specimen, overall architecture (lobular or diffuse), the extent of vascular proliferation (focal or diffuse), and the presence of epithelioid endothelial cells were evaluated. Furthermore, the characteristic features of the dermal inflammatory infiltrate were assessed, including distribution (perivascular, band-like, periadnexal, or diffuse), depth (papillary dermis, reticular dermis, or subcutis), predominant cell type (lymphocytes, eosinophils, or plasma cells), and presence/absence of germinal centers. All specimens were immunohistochemically stained using the three-step indirect peroxidase complex technique after a preliminary automated pressure-based antigen retrieval step. Diaminobenzidine was then applied as the chromogen. All specimens were stained with anti-Ki-67, anti-CD34, anti-CD45, anti-CD19, anti-CD20, anti-PAN-CK, anti-Cd1A, anti-desmin (D33) (1:320, Pathnsitu), anti-smooth muscle actin (anti-SMA, 1:160, Pathnsitu), anti-keratin cocktail (AE1/AE3, 1:1280 Pathnsitu), anti-factor VIIIrAg (1:5120, Dako), anti-CD31(1:80, Dako), and anti-CD34 [1:640, m(QBEnd/10),Dako] monoclonal antibodies.

**Figure 1:**
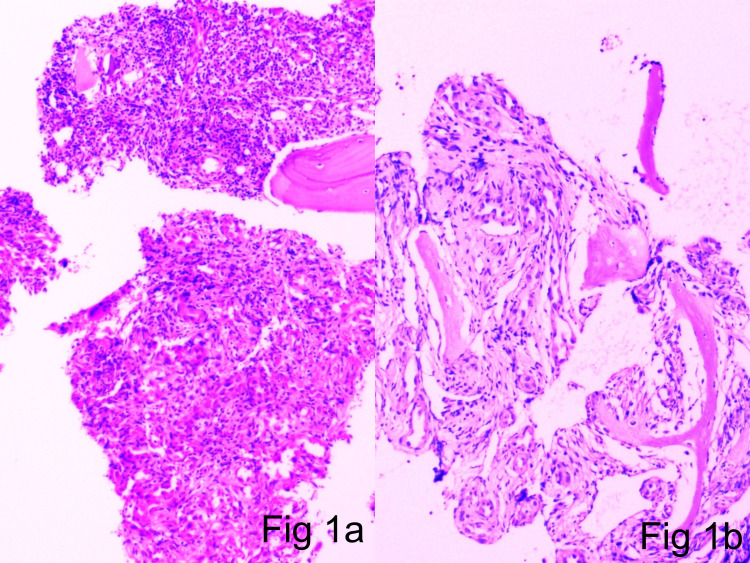
1a and 1b: Cortical and trabecular bone with an infiltrative vascular neoplasm (H&E scanner view) H&E: hematoxylin and eosin

The first patient in the case series was a 15-year-old male. He presented in the casualty with a slowly growing swelling in the forefoot for two years. The X-ray revealed an ill-defined osteolytic lesion in the second metatarsal bone with expansile margins. A provisional diagnosis of enchondroma was made. The patient was treated with bone curettage. Microscopy revealed an irregular infiltrative vascular neoplasm with a nodular/lobular growth pattern without any demonstrable vessel origin (Figure 1).

**Figure 2:**
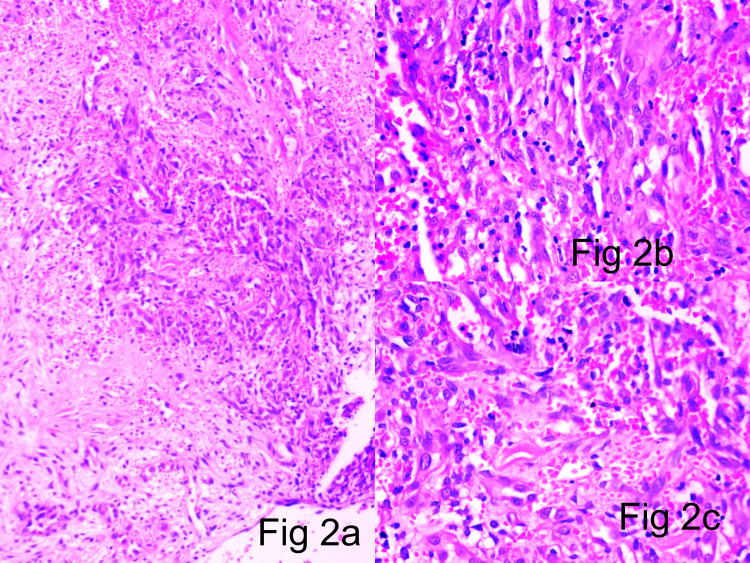
2a (H&E x10), 2b (H&E x40), 2c (H&E x40): Central solid areas composed of endothelial cell sheets with interspersed slit-like channels H&E: hematoxylin and eosin

The pattern of growth in the central zone was diffuse in sheets. Focal spindling of cells arranged in fascicles with slit-like vasculature was noted (Figure 2). Peripherally, the pattern of growth showed well-formed vascular channels with prominent epithelioid endothelial cell morphology and hobnailing.

The tumor-associated stroma showed sheets of eosinophils, along with lymphocytes, extravasated red blood cells (RBCs), and plasma cells. Lymphoid aggregates were seen but were negative for germinal centers. The growth pattern was diffuse and extending into the deep dermis of the overlying skin. A diagnosis of EH involving the second metatarsal bone was made. There was no recurrence on follow-up. The patient was declared cured, and no subsequent treatment was given on follow-up.

**Figure 3:**
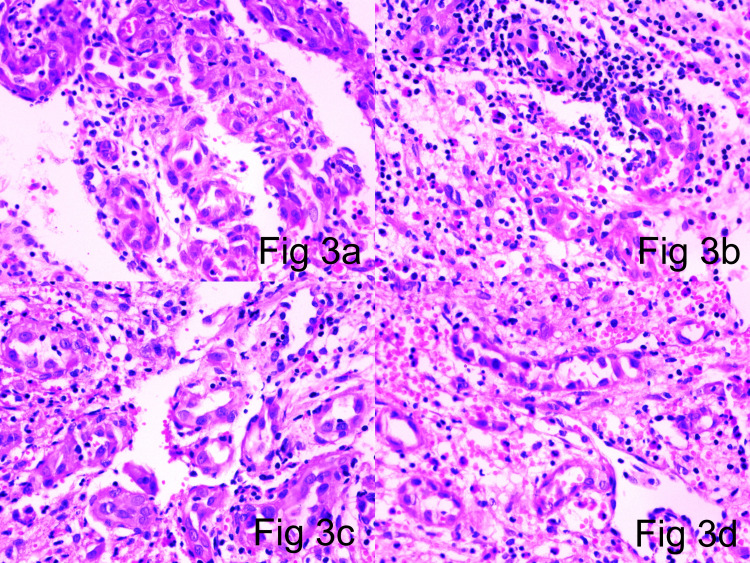
3a (H&E x10), 3b (H&E x10), 3c (H&E x10), 3d (H&E x10): Peripheral well-defined vasformative areas showing large vessels with plump hobnailed endothelial cells and epithelioid endothelial cells H&E: hematoxylin and eosin

The second patient in the case series was a 25-year-old female. The patient presented in the OPD with an insidious onset and progressive swelling in the wrist. X-ray showed an ill-defined osteolytic lesion involving the carpal bones. The lesion also showed cortical erosion and periosteal reactive bone formation. A clinical provisional diagnosis of an aneurysmal bone cyst was made. The patient was treated with bone curettage. Microscopy showed a nodular/lobular growth pattern of a vascular neoplasm without any demonstrable vessel origin. The central growth pattern was in diffuse sheets. Focal spindling of the cells was noted, which were arranged in fascicles with slit-like vascularity. Peripheral well-formed blood vessels were seen with epithelioid endothelial cells showing prominent hobnailing (Figure 3).

**Figure 4:**
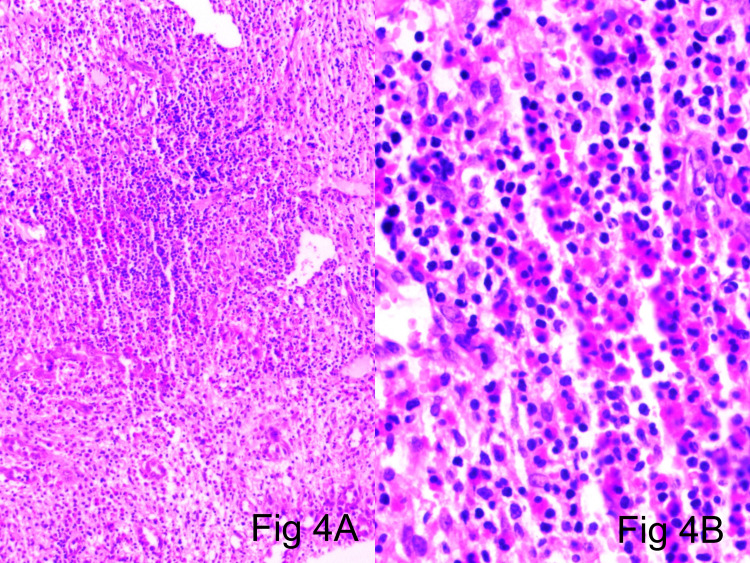
4a (H&E scanner), 4b (H&E x40): Tumor-associated stroma showing sheets of eosinophils along with lymphocytes and plasma cells H&E: hematoxylin and eosin

The tumor-associated stroma showed sheets of eosinophils, eosinophilic microabscesses, along with lymphocytes and plasma cells. Focal lymphoid aggregates were seen but were negative for germinal centers (Figure 4).

There was focal spindling of cells arranged in fascicles with slit-like vasculature. A diagnosis of EH involving the carpal bones was made. There was no recurrence on follow-up. The patient was declared cured and no subsequent treatment was given on follow-up.

**Figure 5:**
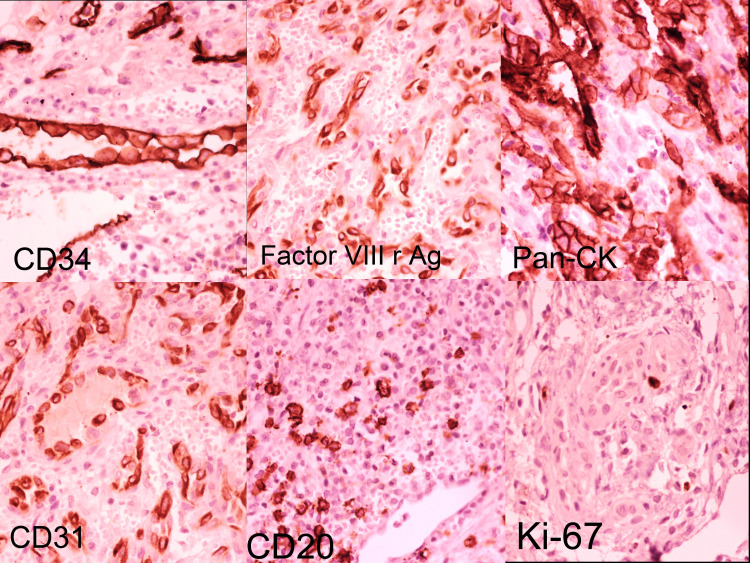
Tumor-associated epithelioid endothelial cells showing immunohistochemical positivity for CD34, Factor VIIIr and Ag, PAN-CK, as well as a low Ki-67 mitotic index CD: cluster of differentiation; PAN-CK: pan-cytokeratin

The third patient in the case series was a 20-year-old male. The patient presented with an osteolytic lesion in the first metatarsal bone. The patient presented in the casualty with a progressive slowly growing mass, which was insidious in onset. Radiologically, the lesion was ill-defined, expansile, with sclerotic margins and reactive bone formation. A clinical provisional diagnosis of enchondroma was made. The patient was treated with bone curettage. Microscopy showed a nodular growth pattern without any demonstrable vessel origin. The central growth pattern was diffuse in sheets with peripheral, well-formed channels. The vessels showed prominent epithelioid endothelial cells with hobnailing. The tumor-associated inflammatory stroma showed sheets of eosinophils along with lymphocytes and plasma cells. The growth pattern was diffuse and extending into the deeper dermis of the overlying skin. The epithelioid endothelial cells were immunoreactive for cluster of differentiation 31 (CD31), CD34, factor VIIIrAg, and pan-cytokeratin (PAN-CK). Smooth muscle actin, desmin, and muscle-specific actin (HHF-35) (Figure 5) were expressed in all cases and highlighted a population of myopericytic cells intimately associated with epithelioid endothelial cells. The cells were negative for CD1a, langerin, and CD86.

A diagnosis of EH involving the first metatarsal bone was made. There was no recurrence on follow-up. The patient was declared cured and no subsequent treatment was given on follow-up.

The clinical characteristics, including the patients’ demographics, lesions, treatment, and follow-up, are summarized in Table 1.

**Table 1:** Histopathological features of three bone epithelioid hemangioma cases OPD: outpatient department

Case	Age	Sex	Location	Clinical Setting	Radiology	Clinical Impression	Treatment and Follow-Up	Overall Architecture	Pattern of Vascular Proliferation	Presence of Epithelioid Endothelial Cells	Predominant inflammatory Cell Component and Distribution	Distribution and Depth of Inflammation	Molecular Study
1	15	M	Second metatarsal	Casualty patient with slowly growing swelling in the forefoot for 2 years.	Ill-defined osteolytic lesion with expansile margins.	Enchondroma	Patient treated with bone curettage and there was no recurrence on follow-up. Patient was declared cured and no subsequent treatment was given on follow-up.	Nodular lobular pattern without demonstrable vessel origin.	Central diffuse growth in sheets. Peripheral well-formed blood vessels. Focal spindling of cells arranged in fascicles with slit-like vasculature.	Well-formed vessels toward the periphery showing epithelioid endothelial cells with prominent hobnailing.	Eosinophils along with lymphocytes and plasma cells.	Diffuse and extending into the deep dermis of the overlying skin.	Not done due to patient’s disinterest.
2	25	F	Carpal bone	OPD patient with insidious onset and progressive swelling in the wrist.	Ill-defined osteolytic lesion with cortical erosion and periosteal reactive bone formation.	Aneurysmal bone cyst	Patient treated with bone curettage and there was no recurrence on follow-up. Patient was declared cured and no subsequent treatment was given on follow-up.	Nodular lobular pattern without demonstrable vessel origin.	Central diffuse growth in sheets. Peripheral well-formed blood vessels. Focal spindling of cells arranged in fascicles with slit-like vasculature.	Well-formed vessels toward the periphery showing epithelioid endothelial cells.	Eosinophils along with lymphocytes and plasma cells.	Diffuse.	Not done due to patient’s disinterest.
3	20	M	First metatarsal	Casualty patient with progressive insidious onset and slowly growing swelling in the forefoot for 1 year.	Ill-defined expansile sclerotic lesion with reactive bone formation and periosteal reaction.	Enchondroma	Patient treated with bone curettage and there was no recurrence on follow-up. Patient was declared cured and no subsequent treatment was given on follow-up.	Nodular lobular pattern without demonstrable vessel origin.	Central diffuse growth in sheets. Peripheral well-formed blood vessels.	Well-formed vessels toward the periphery showing epithelioid endothelial cells with prominent hobnailing.	Eosinophils along with lymphocytes and plasma cells.	Diffuse and extending into the deep dermis of the overlying skin.	Not done due to patient’s disinterest.

Pathological features of the case series

Microscopic examination of tumor specimens revealed an ill-defined lobular contour and no visible symmetrical arterial/parent feeder vessel association in all three cases. The vascular components exhibited central exuberant solid sheets of endothelial cells interspersed by slit-like channels containing red blood cells, with well-defined vessels in the lesion periphery compared with that in the central zone (Figure 1a-1b, Figure 2a-2b). Larger blood vessels were lined by well-defined epithelioid endothelial cells showing hob nailing within foci (Figure 3). Mitotic figures were extremely infrequent, with only 1-5 mitoses/10 high-powered field (hpf). The stroma was densely fibrocollagenous, showing eosinophils sheets along with lymphocytes and plasma cells (Figure 4).

There was no prominent germinal center formation in any sample. The epithelioid endothelial cells were immunoreactive for CD31, CD34, and factor VIIIrAg. Smooth muscle actin, desmin, and muscle-specific actin (HHF-35) (Figure 5) were expressed in all cases and highlighted a population of myopericytic cells intimately associated with epithelioid endothelial cells. The cells were negative for CD1a, langerin, and CD86. All lesions lacked extensive mitotic activity, atypical mitotic figures, and nuclear atypia. There was no prominent germinal center formation in any sample. The epithelioid endothelial cells were immunoreactive for CD31, CD34, and factor VIIIrAg. Smooth muscle actin, desmin, and muscle-specific actin (HHF-35) (Figure 5) were expressed in all cases and highlighted a population of myopericytic cells intimately associated with epithelioid endothelial cells. The cells were negative for CD1a, langerin, and CD86. All lesions lacked extensive mitotic activity, atypical mitotic figures, and nuclear atypia.

## Discussion

Historic classification of bone EHs

Numerous theories on EH tumorigenesis have evolved since its discovery as a distinct pathological entity in the 1960s. EHs are now defined as lesions of distinct endothelial phenotype and epithelioid morphology. Although EH is considered a benign entity, it has metastatic potential. Hartmann and Stewart (1962) provided one of the first detailed descriptions of bone hemangioendothelioma from a case series at Memorial Sloan-Kettering Cancer Center and emphasized its unexpectedly favorable clinical course for malignant vascular tumor [5]. In 1979, Rosai et al. proposed a unifying disease model encompassing several previously described diseases of the skin, soft tissue, large vessels, bone, and heart [6]. The histologic similarity of at least a subset of lower-grade hemangioendothelioma of bone cases to angiolymphoid hyperplasia with eosinophilia (ALHE) suggests that both are representative of a single neoplastic but benign entity subsequently named “histiocytoid hemangioma.” Weiss and Enzinger introduced the term soft-tissue EHE to describe a borderline to a low-grade biologically malignant tumor that was histologically similar to but less aggressive than EAS [7]. Although Weiss and Enzinger were doubtful about unifying the lesions included under the umbrella of histiocytoid hemangioma, they concurred that ALHE was neoplastic and suggested the term “epithelioid hemangioma” [7]. Many pathologists accepted Weiss and Enzinger’s newly proposed nomenclature; EHEs were subsequently described in multiple other sites, including the bone, lung, and liver [8]. In early review articles, O’Connell et al. [9] and Wenger and Wold [10] proposed classifying vascular neoplasms of the bone along the same lines used for skin and soft-tissue pathologies such as EH, EHE, and EAS. Floris et al. proposed that EH, although usually benign, is a potentially metastasizing lesion; however, the authors stressed that this rare occurrence does not justify upgrading EH into the malignant category [11]. Nonetheless, considering the occasional reported cases of recurrence and lymph node involvement, in the most recent 2020 WHO classification, EH is classified as intermediate grade and EHE and EAS as malignant. Therefore, it is important to accurately distinguish EH from EHE and EAS owing to these critical differences in clinical behavior and prognosis [11].

Occurrence sites

When EH occurs in osseous tissues, it is most frequently found in the metaphysis and diaphysis of long tubular bones of the extremities, followed by the short tubular bones of the distal lower extremities and flat bones. Males and females aged 30-60 years are equally affected. Patients usually present with insidious onset, slow-growing soft-to-bony swellings, with relevant joint-related movement impairment in the extremities. Occasionally, the clinical presentation can be a pathological fracture that can occur secondary to osteolysis in these lesions. The anatomic distribution of soft-tissue EHs is broad and the most frequently affected sites are the head, particularly the forehead, preauricular area, and scalp, often in a superficial temporal artery distribution. Tumors have also been documented in the extremities (arm, triceps, hand, tibia, and foot) and less frequently in the trunk (rib, vertebra, axilla, neck, and clavicle). Lesion sites in soft tissue include the lacrimal gland [12], inner canthus [13], heart [14], eye [15], penis [16], scrotum [17], testis [18], colon [19], oral mucosa [20], arteries of the limbs [21], bone [4], lymph nodes [22], lung [23], tongue [24], breast [25], and spleen [26].

Radiologic findings

**Figure 6:**
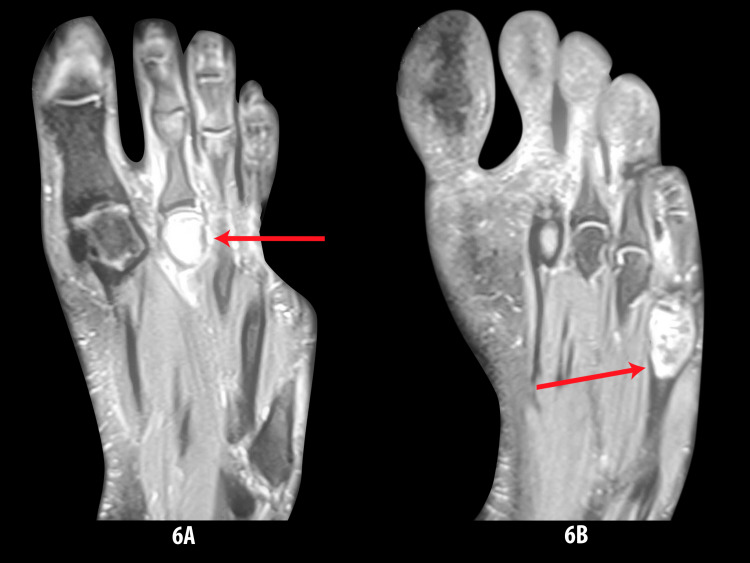
6A and 6B: Radiology showing osteolytic and sclerosing lesions in metatarsal bone

X-ray imaging is generally ineffective for bone EH diagnosis because there are no distinguishing radiologic features. On conventional X-rays, the lesions are usually lucent with well-defined expansile margins [4]. They can also show a mixed lytic and sclerotic appearance with septations, partial cortical destruction, and thick periosteal reactive bone formation (Figure 6) [10].

However, differential diagnoses based on these plain radiographic features are non-specific and can include giant cell tumor, aneurysmal bone cyst, brown tumor, infectious spondylitis, and metastatic deposits. Similar to plain X-ray, computed tomography (CT) scans of bone EH show well-defined, septate, expansile, lytic lesions with cortical destruction and bony expansion. Bone EH is well-defined by MRI, being hypointense or isointense relative to muscle on T1-weighted images, and hyperintense on T2-weighted images. Lesions are markedly enhanced by gadolinium contrast, but even non-contrast MRI reveals well-defined lesions that are isointense or slightly hyperintense relative to skeletal muscle on T2-weighted images. There is variable surrounding marrow edema and enhancement. Cortical disruption with periosteal reaction can also be seen. Moreover, similar imaging manifestations, including multifocal presentation, aggressive radiologic appearance, and/or lymph node metastasis, may be present in both benign and malignant types; therefore, it should not be considered definitive for the differential diagnosis [2,10,27].

Molecular events and pathogenesis

The ALHE/EH etiology is currently unclear. Various hypotheses have been established, including a reactive process [27], a neoplastic process [28], and infectious mechanisms, including a possible association with human immunodeficiency virus (HIV) [29]; however, none are conclusive. EH may arise from an unusual reactive process following local trauma [27], infection [29], arteriovenous shunting [30], or hyperestrogenemia [31]. Further trauma by inciting cellular proliferation may “open the door” for a cytogenetic event [32], albeit one with limited biological (proliferative/growth) potential. Recent studies have demonstrated distinct molecular cytogenetic events contributing to tumorigenic pathways in EH and EHE. FOS gene fusion involvement could be a highly specific EH driving event because fusion events have been identified in one-third of cases across multiple anatomic locations (but with greater prevalence in intraosseous locations). Furthermore, ZFP36-FOSB fusion was identified in a subset of EH cases with atypical histological features by FOSB immunohistochemical expression [32]. Furthermore, distinctive WWTR1-CAMTA1 and YAP1-TFE3 gene fusions have been identified in EHE but not in other epithelioid vascular tumors [33,34]. These findings identify objective molecular tools for distinguishing EH from EHE, which is of paramount importance considering the greater aggression of the latter. However, a subset of cases of pseudomyogenic haemangioendothelioma harbors a t(7;3)(q22;q13) translocation that also leads to the SERPINE1-FOSB gene fusion [35]. SERPINE1 encodes the plasminogen activator inhibitor-1, which is highly expressed in endothelial cells. FOSB fusions are also found in a subset of EH, and although they do not show significant morphologic overlap with PMH, this lends credence to the idea that FOSB oncogenic activation is an important event in some benign and intermediate-grade vascular neoplasms.

Cellular events and pathogenesis

A cause-and-effect relationship has been suggested between trauma and reparative endothelial proliferation. Traumatic damage to vessel walls exposes endothelial cells to excessive arterial pressure and inflammation-associated cytokines, causing these cells to proliferate and acquire a round or oval epithelioid morphology with abundant eosinophilic cytoplasm [36]. The cells also develop cytoplasmic vacuoles representing the earliest stage of vessel lumen formation; their fusion results in vascular spaces with varying degrees of differentiation [4]. Proliferation and further differentiation of these cells result in vessel formation to constrain or divert the arterial pressure. These newly formed vessels show hob nailing of luminal cells. As lesions mature, they exhibit symmetric association with muscular arteries and increased peripheral maturation over time.

Microscopic features

Accurate assessment of lesional microscopic features and IHC expression profile is of paramount importance for the differential diagnosis of these epithelioid vascular neoplasms. The morphologic features of these tumors depend on the formation stage and soft tissue/bone location as well as on the presence and nature of vascular and inflammatory components. In all types, neoplastic cells are of endothelial immune phenotype. Typical variants are mature lesions with well-defined vasoformative tendency and vessel formation increasing from the center to the periphery [36]. At the center of the lesion, solid sheets of endothelial cells are observed (Figures 1-2), whereas the periphery may contain fully canalized vessels with a defined smooth muscle coat and epithelioid endothelial cell lining (Figures 3a, 3c). The epithelioid endothelial cells constituting the neoplasm are generally large and polyhedral, often contain abundant dense eosinophilic cytoplasm, and have a hobnail morphology in the lumen (resulting in a tombstone pattern, see Figures 3b, 3d) [2]. In some tumors, however, the cytoplasm has a finer, feathery, or foamy vacuolated appearance, resembling that of histiocytic cells. Cytologic atypia is usually mild, but occasional cell foci with moderate nuclear pleomorphisms are observed, including multilobular nuclei or pseudo-nuclear inclusions. In addition to this general absence of cytologic atypia, these lesions show little mitotic activity or necrosis, with mitotic figures typically found at <1/10 hpf and always of normal structure [2,37]. Although most tumors are purely epithelioid, large spindling areas containing short fusiform or oval bland endothelial cells are also observed (Figures 2a-2c) [2,38]. Hence, focal spindling and abundant hemorrhage are common in EH (Figures 2b-2c) and thus cannot be used as exclusion criteria. The term “hemangioendothelioma, not otherwise specified” of the bone is a “catch-all” diagnosis and should be avoided where possible [2,38-39]. Other more infrequent ﬁndings include scattered intratumoral osteoclast-type giant cells and reactive bone formation that can compartmentalize the tumor into small nodules. In such cases, the tumor stroma comprises loose connective tissue and a prominent inﬂammatory inﬁltrate rich in eosinophils (Figure 4), lymphocytes, and plasma cells [40-44].

Subtle histomorphologic differences exist among EH of the skin, dermis, and bone, as summarized in Table 2.

**Table 2:** Histological features of epithelioid hemangioma (EH) in different tissues

	EH of the skin and subcutaneous tissue	EH of soft tissue	EH of the bone
Neoplastic potential of the vascular component	Reactive lesion to trauma. Angiocentric distribution around a larger vessel with evidence of mural damage often associated with trauma.	Reactive lesion to trauma. However, a case of dermal EH was found to harbor a TEK gene mutation, which encodes the endothelial cell tyrosine kinase receptor Tie-2, indicating that certain molecular abnormalities may also contribute to pathogenesis [41].	Distinct cytogenetic events and lack of an eosinophilic response in some tumors suggest distinct pathogenesis compared with that in skin EH. FOS gene rearrangement and recurrent ZFP36–FOSB fusion are present in nearly one-third of bone EH cases in varied locations [32].
Neoplastic potential of the inflammatory cell component	These lesions are associated with various lymphoproliferative conditions, supporting the contention that some EH cases arise from a monoclonal T-cell process [42].Peripheral T-cell lymphoma was reported in a patient with ALHE/EH. Some cases of ALHE/EH have also been reported with T-cell receptor gene rearrangement and monoclonality [42].		Mostly polyclonal and reactive. No lymphoproliferative conditions documented to date.
Margins	Well-marginated lesions.	Less-marginated lesions [33].	Less-marginated lesions.
Association with an artery	Demonstrable angiocentric distribution and symmetric association with an artery. The artery can show evidence of damage (e.g., thrombosis, fibrointimal proliferation, duplication of the internal elastic lamina, or mural disruption) [43].	Rarely associated with a muscular artery [33].	Symmetrical association with an artery is not usually demonstrable in bone lesions because the site of origin may be obliterated by the expanding tumor [2].
Vasoformative tendency	Vasoformative tendency and vessel maturation increase from the center to the periphery, resulting in central ill-defined poorly formed vessels (Fig 2) and peripheral well-formed vessels (Fig 3). The subcutaneous form has a tendency for the florid proliferation of large epithelioid endothelial cells that may become so exuberant as to form solid intraluminal nodules or clusters. These masses can obscure the vascular nature of the lesion and thus increase the diagnostic complexity [44-45].	Similar vasoformative tendency to EH of the skin but lesions contain more fully developed vessels, typically with patent lumina. [33].	Vasoformativetendency but often greater histological variability within lesions.
Histomorphology	Well-defined epithelioid endothelial morphology (Figure 3).	Less pronounced epithelioid endothelial morphology (often more cobblestone-like) [36].	Recognizable epithelioid endothelial morphology. Spindling and fasciculation may be observed within the lesion [37].

Further, bone EH shows a greater degree of histologic variability than skin and soft-tissue EH [2]. Depending on the EH developmental stage, vascular or inflammatory components may predominate. In early or actively growing EHs, the vascular component predominates, whereas, in late stages, lymphocytes become more prominent. The vessels in early ALHE/EH are immature with prominent epithelioid endothelial cells; however, when the lymphoid infiltrate predominates in the later stage, endothelial cells lining the maturing vessels become smaller and less epithelioid. Confounding histomorphologic features of vascular and inflammatory components.

When the Vascular Component Predominates

The vasoformative tendency of the vascular component generally decreases from lesion center to periphery. The central indeterminate solid component can predominate in exuberant or atypical variants. The differential diagnoses in such cases are EHE [9], EAS [41], and very rarely, Kaposi sarcoma. Detailed histological evaluation is critical in such equivocal cases to prevent overly aggressive intervention. A number of histological parameters must be assessed (Table 3), and if the sample size is too small to establish a diagnosis with confidence, additional sampling should be requested.

**Table 3:** Clinical and histological features distinguishing EH, EHE, and EAS EH: epithelioid hemangioma; EHE: epithelioid hemangioendothelioma; EAS: epithelioid angiosarcoma

Histomorphological features	EH		EHE	Pseudomyogenic hemangioendothelioma	EAS
Neoplastic nature	Benign with metastasizing potential [46].		Intermediate [46].	Intermediate [47].	High-grade [4].
Association with artery	Intimately associated with and symmetrically distributed with a small muscular artery.		Lacks intimate association with a muscular artery [4].Typically affects the veins rather than arteries [4].	Lacks intimate association with a muscular artery [4].	Lacks intimate association with a muscular artery [4].
	Presence of mature vessel foci with open lumen formation [25].		Mature vessels absent [4].	Mature vessels absent [4].	Mature vessels absent [4].
Molecular pathology	FOS gene rearrangement in nearly 1/3 of EH cases in various locations [32].Recurrent ZFP36–FOSB fusion in a subset of EH cases with atypical morphological features that do not suggest FOS gene rearrangement [48]. Recurrent ZFP36–FOSB fusion in a subset of EH cases with atypical histological features. Confirmed FOSB-positive by immunohistochemistry [48]. Negative for WWTR1–CAMTA1 fusion [48].		Recurrent t(1;3)(p36;q25) chromosomal translocation, resulting in WWTR1–CAMTA1 fusion [37].	SERPINE1-FOSB fusion [47].	Complex cytogenetic aberrations. No WWTR1–CAMTA1 fusion or FOS gene rearrangement [37].
Sites	Femur, phalanges, tibia, fibula, metatarsals, scapula, humerus, ilium, vertebrae, sacrum, ribs, and sternum [5].		May involve any bone and has been detected in the femur, tibia, fibula, humerus, radius, ulna, ribs, vertebral bodies, pelvis, scapula, and small bones of the hands and feet [49].	Superficial or deep soft tissue of extremities [47].	Predilection for the femur. Other affected bones include the tibia, calcaneus, humerus, radius, and small bones of the hand, rib, and pelvis [50]
Multifocality	Unicentric to multicentric [4].		Unicentric to multicentric [49].	Multicentric [47].	Unicentric to multicentric [50].
Age	Second to eighth decades of life (mean age of 34 years) [20].		Slight male predominance and a wide age distribution extending from the second to eighth decades of life [49].	Males. Young adults. [47]	Striking male predominance with a mean age of 57 years [50].
Gross	Ranges from 2 to 15 cm in the biggest dimension, well-circumscribed, and dark red in color. Mostly confined to the affected bone where they infiltrate the marrow space, surround the bony trabeculae, and abut or erode the cortex. Occasionally, large tumors transgress the cortex and form soft-tissue masses [51].		Ranges from 2 cm to >10 cm. Frequently tan in color and solid in structure. Usually centered in the medullary cavity and grow in an infiltrative and destructive fashion [49].	Ranges from 1 to 2.5cm only with approximately 10% of tumors being >3cm [47]. Grossly, the tumor is ill-defined, usually multifocal, with a white-to-brown cut surface [47].	Usually >5 cm and soft, red, and hemorrhagic. Usually originates in the medullary cavity and invades the cortex and neighboring soft tissues [50].
Radiology	Usually lucent with well-defined expansile margins [20]. In some cases, they show a mixed lytic and sclerotic appearance with septations, partial cortical destruction, and thick periosteal reactive bone formation [6].		Usually lytic or mixed lytic and blastic with well- or poorly defined margins. Can expand the bone contour and elicit a periosteal reaction, especially if a soft-tissue component is present [49].	Usually lytic, lobulated, and well-circumscribed on CT and radiography. On MRI, the lesions are hypointense on T1-weighted images and hyperintense on T2-weighted and stir-weighted images [47].	Predominantly destructive or exclusively lytic, frequently extending into soft tissues.
		Microscopy			
Growth pattern	Lobular growth pattern showing symmetric association with the vessel wall. Infiltrative and destructive but non-metastasizing [10,20].		The lobular growth pattern characterizing EH is absent. No involvement of medium-sized or larger vessels. Infiltrative, destructive, and metastasizing. Higher rates of multifocality and distant spread [4,9-10].		
Tumor architecture	The central areas of the lobules contain epithelioid endothelial cells in solid sheets but without a coherent structure. The periphery shows well-formed vessels [9-10,20].		Primitive epithelioid endothelial cells present singly in cords, clusters, and/or sheet-like arrangements. If spindled, they tend to be oriented in fascicles. Well-formed vascular channels are relatively scant or absent. [4].	Ill-defined nodules and fascicles. Desmoplastic stroma [47].	Irregular vascular spaces, sometimes with a papillary pattern, sheets, and areas of solid growth. May protrude into lumens of well-developed vascular spaces in the form of solid tufts or nodules.
Vasoformative tendency	Vasoformative tendency and vessel maturation increase from the center to the periphery. [9,10,20].		Frequent intracytoplasmic lumens.	Not present [47].	Vascular channels. Intracytoplasmic lumens [52]
Tumor cell morphology	The cytoplasm is abundant, eosinophilic, and frequently contains one or more large clear vacuoles (Figure 3).		Populations of elongated and spindle-shaped cells. Elongated cells have prominent intracytoplasmic vacuoles, whereas vacuoles are rare in spindle cells.	Plump spindle or epithelioid cells, with abundant brightly eosinophilic cytoplasm, sometimes mimicking rhabdomyoblasts [47].	Numerous intracytoplasmic vacuoles [52].
Nuclear morphology	Consistent absence of anaplasia, cytologic atypia, and necrosis. Minimal necrotic activity.		The tumor cells contain vesicular nuclei, often with small nucleoli. Greater variability in size and degree of nuclear atypia than with EH. Differentiation from EAS may be difficult if atypia is severe.	The tumor cells contain vesicular nuclei, often with small nucleoli. The degree of nuclear atypia is usually mild, and mitotic activity is scarce [47].	Marked nuclear atypia and pleomorphism.
	Usually <5 mitoses per 10 hpf. Mitotic figures are structurally normal [1].		Greater atypia than EH;>5 mitoses per 10 hpf [1].	Greater atypia than EH;>5 mitoses per 10 hpf [47].	Abundant atypical mitotic figures (>20 per hpf) [1].
Tumor-associated inflammation and stroma	The stroma contains loose, fibrous connective tissue that often contains lymphocytes, varying numbers of eosinophils (Fig 4), and sometimes numerous extravasated red blood cells.		Little inflammatory infiltrate or peritumoral reactive change, unlike EH.	Approximately 50% of cases contain prominent stromal neutrophils, unlike epithelioid hemangioendothelioma (EHE) and angiosarcoma.	Neutrophilia and diffuse interstitial hemorrhage are common and necrosis may be extensive. Little lymphoplasmacytic infiltrate or eosinophilic infiltrate, unlike EH.
	No stromal hyalinization or a basophilic ground substance resembling the cartilage (a feature characteristic of EHE). Reactive woven bone is commonplace and when abundant can mimic a bone-forming neoplasm.		Characteristically, stroma has a hyalinized or basophilic appearance and lacks inflammatory cells. [44,53-54]. This finding is associated with the epithelioid appearance of tumor cells frequently resulting in EHE being mistaken as metastatic carcinoma or a primary cartilage neoplasm.	Occasionally contain focally myxoid stroma [47].	Hyalinized or basophilic stroma is absent. Myxohyaline stroma can be a focal feature. Post-radiation specimens may show prominent fibrosis and hyalinization.
IHC for muscle-specific actin	Zonation pattern [33].		No zonation pattern.	No zonation pattern.	No zonation pattern.
Other IHC markers and their predictive values.	CD34^+^, CD31^+^, Fli-1^+^, ERG^+^, FOSB ^+^, CKs^+^, CAMTA1-, INI-1 retained. (100% CAMTA1, TFE3 negative 21% FOSB-positive) [47].		CD34^+^, CD31^+^, Fli-1^+^, ERG^+^, FOSB -, CKs^+^, CAMTA1^+^, INI-1 retained. (100% FOSB-negative, 62% CAMTA1-positive-TFE3 negative, 38% CAMTA1-negative, TFE3-positive) [47].	CD34-, CD31^+^, Fli-1^+^, ERG^+^, FOSB^+^, CKs^+^, CAMTA1 -, INI-1 retained. (100% CAMTA1, TFE3 negative, 100% FOSB-positive) [47].	CD34^+^ (50%), CD31^+^, Fli1^+^, ERG^+^, FOSB -, CKs^+^, CAMTA1 -, INI-1 retained. (100% -CAMTA1,TFE3-negative, FOSB-negative) [47].
Treatment	Depending on the size and location of the lesion(s), curettage, conservative en block excision, or wide resection is recommended.		The number, size, location, and stage of the tumor determine the treatment type. Wide resection is recommended when feasible. Chemotherapy has been suggested for widespread multisystemic involvement.	The treatment of choice has been surgery (wide resection), with certain cases receiving radiotherapy or chemotherapy. Recently, high expression of mTOR has been demonstrated by immunohistochemistry, and two cases treated with mTOR inhibitors showed clinical improvement [7, 36].	Wide surgical resection with or without adjuvant radiation and chemotherapy. These tumors have an extremely high rate of metastasis and virtually all patients die within 1–2 years of diagnosis.

As one factor for distinguishing possible EH, both EHE and EAS less frequently involve the bone [2]. Although once described as distinct entities, hemorrhagic epithelioid and spindle cell hemangioma are now considered EH variants [37]. The typical nodular EH variants can resemble pyogenic granuloma, which comprises tight aggregates of capillary-sized vessels growing in a lobular fashion within a fibromyxoid (granulation tissue-like) stroma [45,55]. EH can also exhibit intravascular papillary endothelial cell proliferation; this feature can confound the differential diagnosis from intravascular papillary endothelial hyperplasia (Masson’s tumor) [21]. However, vessels with an irregular lumen and plump epithelioid cells are typically absent in the latter [21]. The differential diagnoses for other cutaneous lesions mainly comprising epithelioid cells include poorly differentiated squamous cell carcinoma, melanoma, epithelioid vascular tumor, atypical fibroxanthoma, cutaneous leiomyosarcoma, epithelioid fibrous histiocytoma, and epithelioid sarcoma [56]. The IHC staining profile should be considered for classifying tumor lineage in such cases.

When the Inflammatory Component Predominates

The predominance of inflammatory components has greater diagnostic implications for soft-tissue lesions. The mixed inflammatory infiltrate is nodular and presents a perivascular and periadnexal distribution. Lymphocytes, histiocytes, eosinophils, mast cells, and plasma cells are scattered [57]. When the mixed inflammatory infiltrate predominates or obscures the vascular component, EH can be misdiagnosed as Kimura’s disease, response to an arthropod bite, or cutaneous lymphoproliferative disorders, among others (Tables 4-6) [57-63]. The key to EH diagnosis in such cases is recognizing the vascular component of the lesion [57].

**Table 4:** Distinguishing EH from Kimura’s disease EH: epithelioid hemangioma

	Epithelioid haemangioma	Kimura’s disease
Ethnicity	Occurs in all ethnicities [57].	Predominantly affects Asian males [57].
Location	Superficial and smaller lesions [57].	Subcutaneous involvement with extension to lymph nodes, underlying soft tissue, and salivary glands [57].
Complete blood counts and serology	Lacks high IgE and eosinophils in peripheral blood [57].	High IgE, eosinophils in peripheral blood, and eosinophilia. Could be misdiagnosed as nephrotic syndrome, asthma, tuberculosis, or Loeffler syndrome [57].
Epithelioid morphology	Epithelioid endothelial cells line all blood vessels [57].	Proliferation of post-capillary venules lined by plump endothelial cells [57].
Overlapping morphologic features	Lymph follicles with germinal center formation and abundant eosinophils (less intense than in Kimura’s disease) [57].	Lymph follicles with germinal center formation and abundant eosinophils (more intense than in EH) [57].
Eosinophilic microabscesses and Charcot–Leyden crystals	Negative [57].	Present [57].

**Table 5:** Distinguishing EH from cutaneous marginal zone lymphoma EH: epithelioid hemangioma

	Epithelioid hemangioma	Cutaneous marginal zone lymphoma
Pattern of inflammation	Nodular polyclonal perivascular or periappendigeal lymphocytic infiltrate with CD30^+^ lymphoid infiltrates [58].	
Added features	Absence of factors listed (right panel) [58].	Dutcher bodies, lymphoplasmacytoid cells, folliculotropism and syringotropism, monoclonality, and aberrant BCL2 expression [58].
Epithelioid vascular component	Prominent epithelioid vascular component [58].	No epithelioid vascular component [58].

**Table 6:** Distinguishing EH from an arthropod bite response and cutaneous epithelioid angiomatous nodules EH: epithelioid hemangioma

	Epithelioid hemangioma	Arthropod bite	Cutaneous epithelioid angiomatous nodules
Extent of capillary proliferation	Marked capillary proliferation [59-63].	Subtle capillary proliferation [59-63].	Sheet-like proliferation of endothelial cells in skin [63].
Growth pattern	Lobular growth pattern with numerous epithelioid endothelial cells [59-63].	No lobular orientation and relatively few endothelial cells [59-63].	No multilobular growth pattern as seen in EH [63].

Metastasis versus multi-centricity of EH

Based on recent findings, EH is now considered a benign vascular tumor with a metastatic potential of monoclonal origin (18%-25% of tumors) [37,64-65]. Van Ijzendoorn et al. provided evidence that multifocal EH results from metastasis of the same neoplastic clone rather than the simultaneous neoplastic formation of multiple EH cell clones [46,54]. Similarly, a case with multifocal EHE of the liver also demonstrated monoclonality [46]. Therefore, multifocal vascular tumors of this type are more likely to be metastatic than multicentric. To date, there have been no reports of fatal EH metastasis, consistent with the current classification [35].

Role of IHC in the differential diagnosis

HHF-35 immunohistochemistry is considered the best available marker (far superior to smooth muscle actin) for confirming the presence of an intact myopericytic layer around immature vessels. The presence of this layer, particularly when its distribution increases from the central to peripheral zones (zonation), is a good indication of lesional maturation. Although this zonation pattern is clearly present in EHE and EAS, it is typically nowhere near as distinct or prevalent as in EH [33]. A small fraction of lesional epithelioid endothelial cells express keratins [38]. This expression can hinder diagnosis because it is a common sign of epithelial neoplasms. However, if attention is paid to the limited extent of the reaction and CD31 and factor VIIIrAg levels are also examined, an incorrect diagnosis of “epithelial neoplasm” can be prevented. Preliminary observations suggest that keratin expression is more frequent in malignant epithelioid vascular tumors than in EH (Figure 5). The other IHC markers that can be used include FLI-1, ERG, FOSB, CAMTA1, TFE-3, and INI-1 [47]. The varying pattern of expression of the above markers among the epithelioid vascular neoplasms helps in the proper subcategorization of the neoplasms, as depicted in Table 3 [47].

Next-generation sequencing and the role of reverse transcription-polymerase chain reaction (RT-PCR) in the classification of epithelioid vascular neoplasms

Immunophenotypic and molecular characterization of these tumors has developed significantly over the last 10 years, as demonstrated in the WHO categorization. A genetic signature of epithelioid hemangioma has been recognized as chromosomal translocation including the FOS gene [32] while rearrangements involving FOSB [47] have been observed in pseudomyogenic hemangioendothelioma and a subset of epithelioid hemangiomas. Such molecular modifications are important diagnostic indicators that can help separate these two tumors from several other vascular tumors. In addition, two novel recurring gene combinations (WWTR1-CAMTA1 and YAP1-TFE3 gene fusions) have been reported for epithelioid hemangioendothelioma [37]. Until now, in all the morphologic mimics of epithelioid hemangioendothelioma, these genetic variations haven't been identified, thereby representing an additional diagnostic instrument. Intriguingly, these mergers contributed to the mutually unique nuclear aggregation of CAMTA1 or TFE3, allowing IHC to be a reliable solution for all variants of epithelioid hemangioendothelioma (Table 3) [47].

Treatment modalities and clinical behavior of EH

Previously, most EHs were conservatively treated, as they were not aggressive [2,66-68]. Indeed, many EH case reports encompass several origin sites and distributions, indicating that this disease does not show disseminated growth that can be construed as aggressively destructive or life-threatening; this supports the concept that bone EH is benign similar to its cutaneous counterpart. The benign nature of this tumor is further supported by reports of spontaneous regression [68]. The simultaneous involvement of multiple organ systems is likely a manifestation of monoclonality with metastatic behavior. Nonetheless, there are no recorded fatalities from metastasis. Although epithelioid bone vascular tumors share only some overlapping morphological and clinical features, they markedly differ in prognosis and recommended management. Based on our experience, we believe that bone EH should be treated with curettage or marginal en bloc excision when appropriate and that this treatment will result in an excellent prognosis [2,4]. However, EHE and EAS should be widely excised and systemic therapy should be considered because these tumors, particularly EAS, can be fatal [2,4]. Thus, it is critical to distinguish EHE and EAS from EH based on morphologic criteria. Moreover, in current advanced molecular diagnostics, one cannot overemphasize the importance of detecting FOS gene rearrangements and recurrent ZFP36-FOSB in the subsets of cases of EH [32]. Recurrent t(1;3)(p36;q25) chromosomal translocation, resulting in WWTR1-CAMTA1 fusion, are seen in a subset of cases of EHE [37]. Judicious usage of these molecular fusion markers can be of immense help in differentiating morphologically close, overlapping cases of EH and EHE. Osseous epithelioid endothelial tumors should be classified into three tiers, analogous to their soft-tissue counterparts, and this approach might help clarify the historic confusion surrounding these entities [2,10,66,69].

## Conclusions

In summary, three cases of bone EH are reported, and the relevant literature describing the classic features distinguishing this disease from other bone epithelioid vascular tumors is summarized in this case series. However, there is no individual early clinical, radiological, or immunohistochemical marker for distinguishing this benign form from other, more aggressive bone vascular tumors. Rather, multiple histologic and IHC parameters must be assessed, and if the sample size is too small to establish a diagnosis with confidence, additional sampling should be requested. It is mandatory to judiciously use ancillary molecular diagnostic techniques for the screening of FOS gene rearrangements and WWTR1-CAMTA1 and YAP1-TFE3 gene fusions for the subtle morphologic differentiation of EH and EHE when necessary.
